# Supplementary material for: Seasonal antigenic prediction of influenza A H3N2 using machine learning
Source: Nat Commun. 2024 May 7;15:3833. doi: 10.1038/s41467-024-47862-9 (PMC11076571; doi:10.1038/s41467-024-47862-9)
Supplement: Supplementary file 1 — Supplementary Information [file 41467_2024_47862_MOESM1_ESM.pdf]

Supplementary information

Seasonal antigenic prediction of influenza A H3N2 using machine learning

Syed Awais W. Shah[1], Daniel P. Palomar[1,2], Ian Barr[3,4], Leo L. M. Poon[5,6], Ahmed Abdul Quadeer[1,7,*], and Matthew R. McKay[4,7,*]

[1]Department of Electronic and Computer Engineering, The Hong Kong University of Science and Technology, Clear Water Bay, Hong Kong SAR, China

[2]Department of Industrial Engineering & Decision Analytics, The Hong Kong University of Science and Technology, Clear Water Bay, Hong Kong SAR, China

[3]WHO Collaborating Centre for Reference and Research on Influenza, Melbourne, Victoria, Australia

[4]Department of Microbiology and Immunology, University of Melbourne, at The Peter Doherty Institute for Infection and Immunity, Melbourne, Victoria, Australia

[5]School of Public Health, LKS Faculty of Medicine, The University of Hong Kong, Hong Kong SAR, China

[6]Centre for Immunology & Infection, Hong Kong Science Park, Hong Kong SAR, China

[7]Department of Electrical and Electronic Engineering, University of Melbourne, Melbourne, Victoria, Australia

[*]Corresponding authors:

Ahmed Abdul Quadeer (ahmed.quadeer@unimelb.edu.au)

Matthew R. McKay (matthew.mckay@unimelb.edu.au)

Table of contents

SUPPLEMENTARY FIGURES 2

SUPPLEMENTARY TABLES 12

SUPPLEMENTARY FIGURES

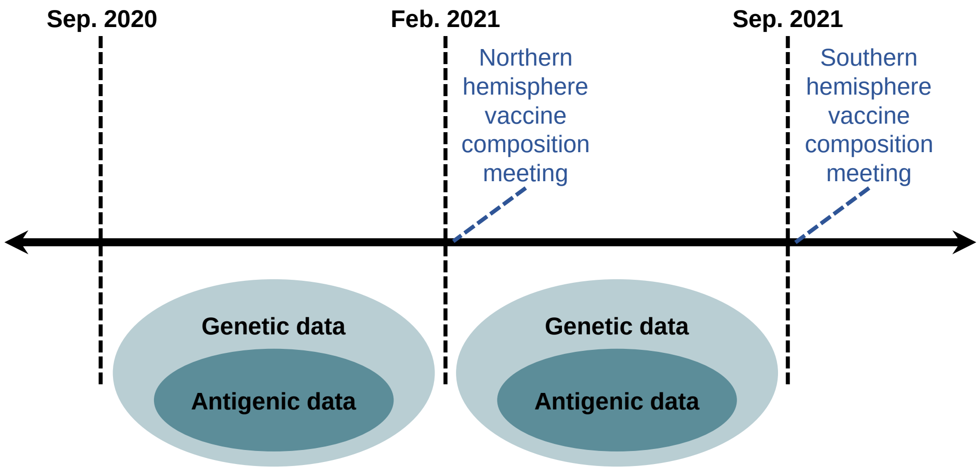

Supplementary Fig. 1 The WHO framework for seasonal antigenic characterization of human influenza viruses. As an example, the framework is shown for the Northern Hemisphere and Southern Hemisphere vaccine composition meetings held respectively in the last week of Feb. 2021 and Sep. 2021. In each season, genetic characterization is performed for most of the circulating isolates. A few representative isolates are then selected for antigenic characterization.

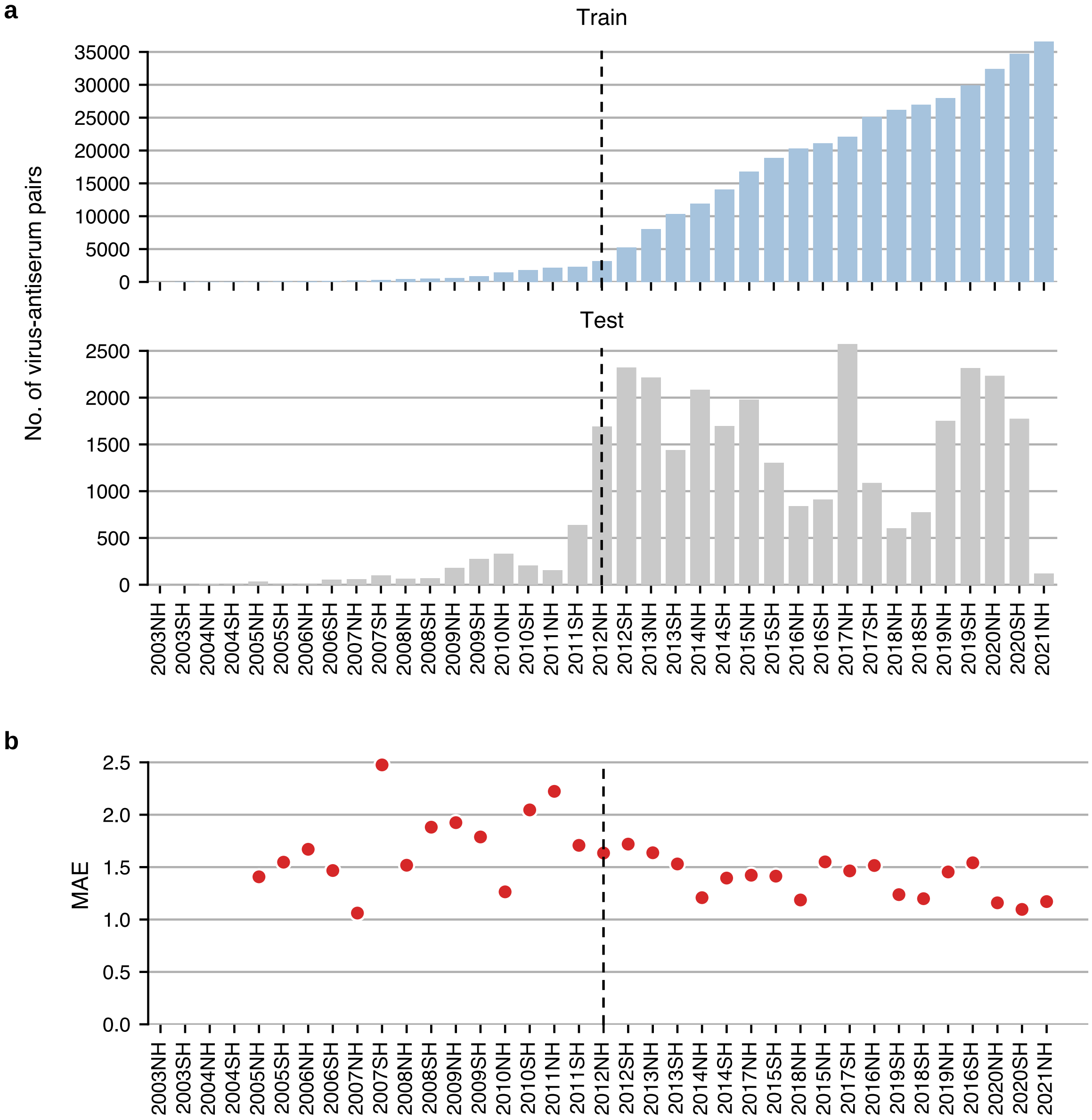

Supplementary Fig. 2 Data distribution and the performance of baseline model over multiple influenza seasons. (a) Under the seasonal framework (**Fig. 1a**), the data is distributed into training and test datasets for each of the 37 seasons from 2003NH to 2021NH. Upper panel shows the number of virus-antiserum pairs in the training dataset for each season, whereas lower panel depicts the same for the test dataset. **(b)** The performance of the baseline model in terms of MAE for each test dataset in 35 seasons from 2005NH to 2021NH. The baseline model is an AdaBoost model with unoptimized hyperparameters, binary encoded genetic difference, and without any metadata information. The vertical dashed line indicates the season after which the baseline model started to provide reliable predictive performance. Source data are provided as a Source Data file.

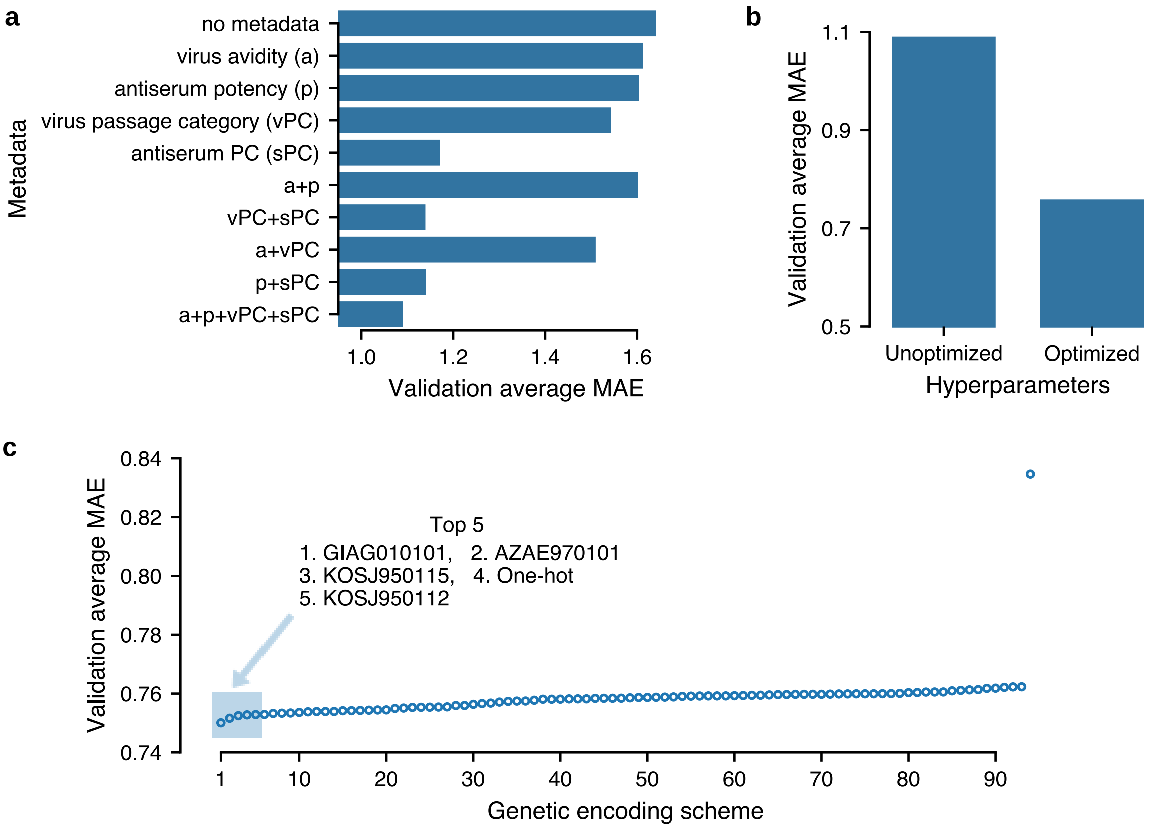

Supplementary Fig. 3 Optimization of AdaBoost model based on metadata information, model hyperparameters, and amino acid attributes. (a) MAE performance of the baseline model when a specific feature or group of features are incorporated in the metadata information. The MAE score was averaged over four validation seasons from 2012NH to 2013SH. **(b)** MAE performance of the baseline model (including all the metadata information) with unoptimized and optimized hyperparameters (see *Methods*). **(c)** Variation in MAE performance of the baseline model (including all the metadata information) over genetic encoding schemes (including the 92 amino acid mutation matrices as well as binary and one-hot encoding). The hyperparameters were optimized independently for each of these 94 AdaBoost models corresponding to mutation matrices and encoding methods. The top five genetic encoding schemes with the best MAE performance are highlighted and listed. Source data are provided as a Source Data file.

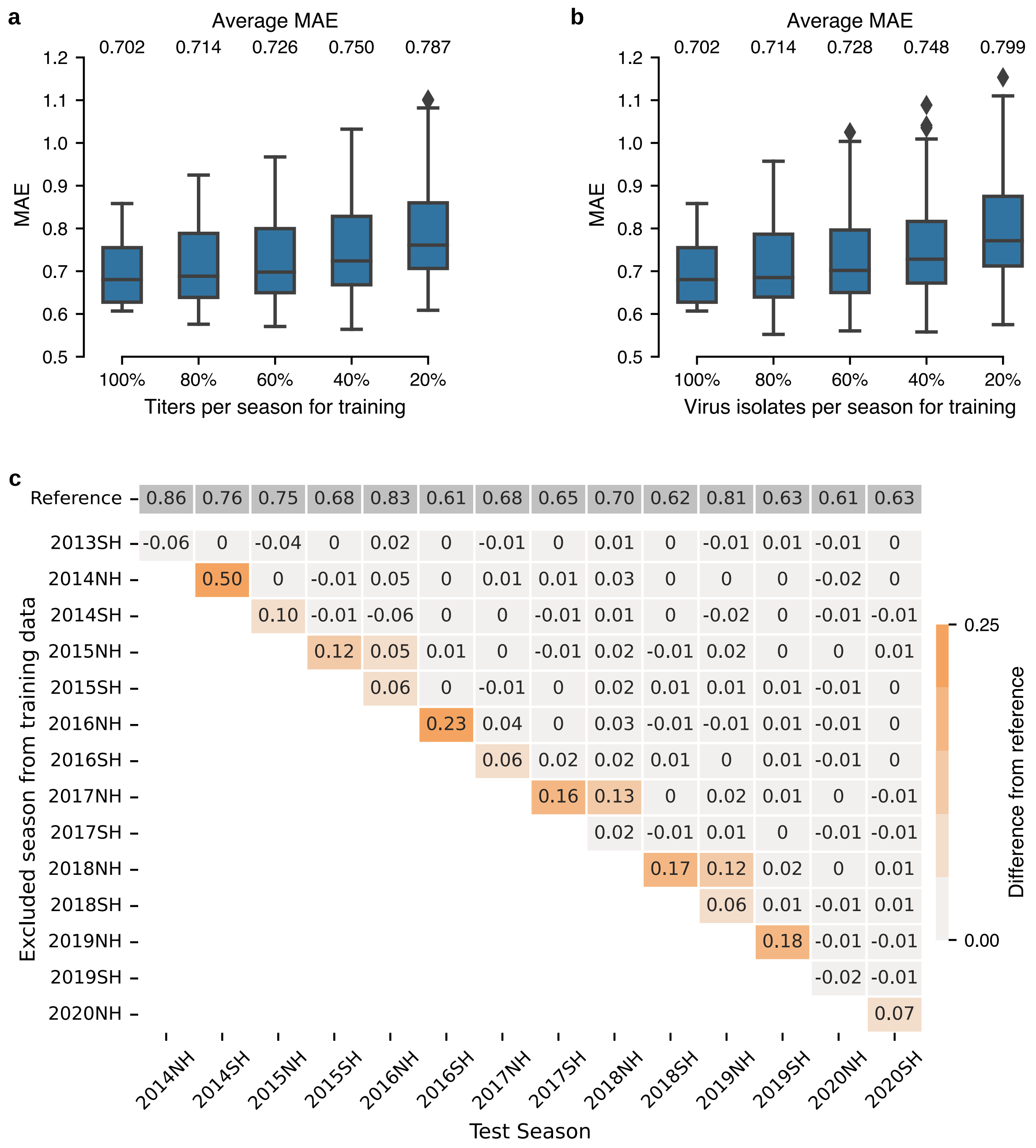

Supplementary Fig. 4 Robustness of the model's predictive capability to changes in training data. (a-b) Performance of models trained over a subset of training data containing **(a)** only 20-80% randomly selected HI titres, or **(b)** all the HI titres of only 20-80% randomly selected virus isolates, in each historical season from 2003NH up to the test season. Each boxplot shows the variation in the MAE performance of the models over 14 test seasons from 2014NH to 2020SH. The average MAE, mentioned over each boxplot, is the average over 50 Monte Carlo runs, where each of these 50 values represent average MAE over 14 test seasons. In each box plot, the middle line indicates the median, the edges of the box represent the first and third quartiles, and whiskers extend to span a 1.5 interquartile range from the edges. **(c)** Performance of the model when training data of the most recent season is excluded from model training. For each test season, 'Reference' indicates the MAE performance of the model trained on the complete training dataset starting from the earliest season 2003NH up to the test season. In the heatmap, each row corresponds to a season excluded from the training data. Each cell in a column shows the change in the MAE performance of the model in comparison to the 'Reference' cell in the same column. The darker cell colour indicates the worse MAE performance. Source data are provided as a Source Data file.

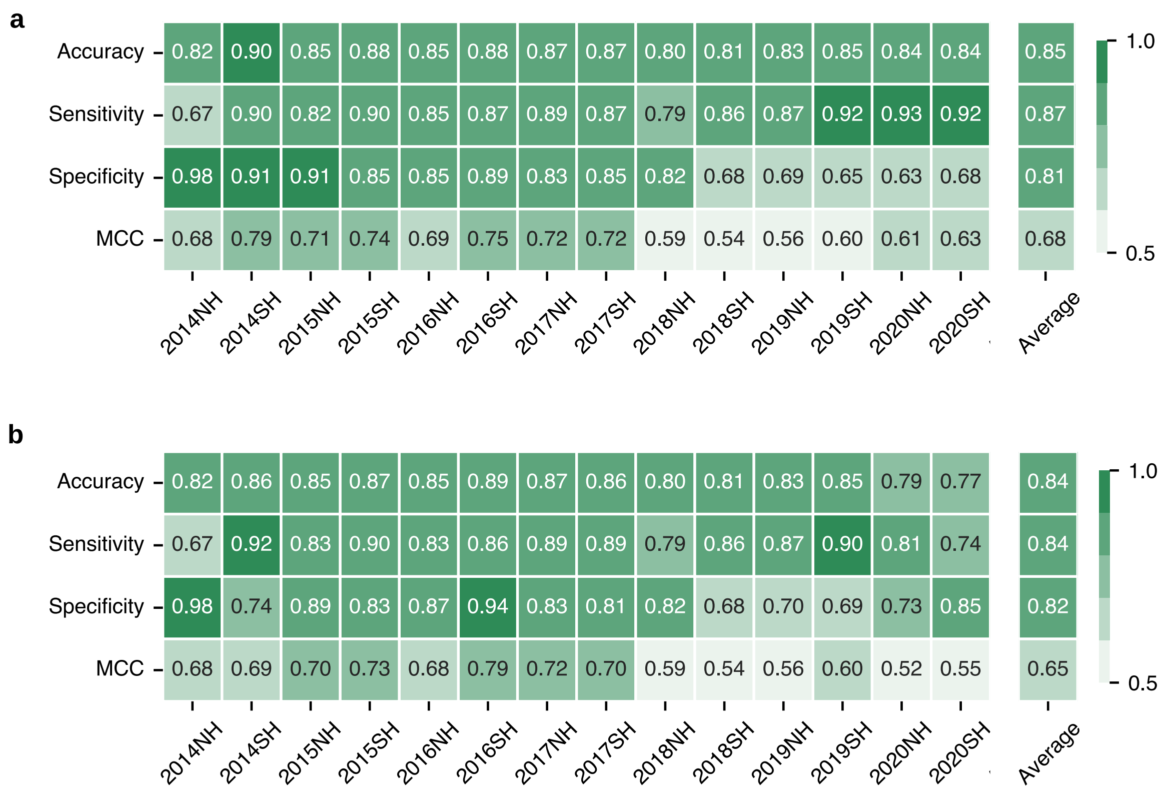

Supplementary Fig. 5 Classification performance of the optimized model. (a) Performance of the model with a classification threshold of 2 antigenic units. The darker cell colour indicates better performance. The 'Average' cell indicates the classification scores averaged over 14 test seasons from 2014NH to 2020SH. **(b)** Performance of the model with an optimized classification threshold. For each test season from 2014NH to 2020SH, the classification threshold was optimized to maximize the Youden's index (sensitivity + specificity – 1) for the previous three seasons. As the Youden's index keeps a balance between the two classes, it therefore decreases the sensitivity and improves the specificity in comparison to the scores in **(a)**. Source data are provided as a Source Data file.

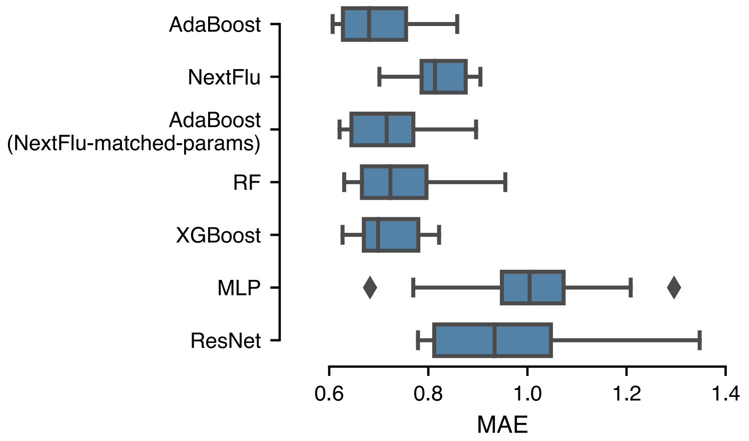

Supplementary Fig. 6 Performance comparison of ML and NN models for antigenic prediction of IAV H3N2 under the seasonal framework. Comparison of the proposed AdaBoost model with a linear model (NextFlu substitution model), tree-based ML models (RF and XGBoost), and NN models (MLP and ResNet). See *Methods* for implementation details of these models. The "AdaBoost (NextFlu-matched-params)" model is based on AdaBoost, with parameters tailored to match those of the NextFlu model that uses binary-encoded genetic differences and only two metadata features: virus avidity and antiserum potency. For each model, MAE was computed for 14 test seasons from 2014NH to 2020SH. In each box plot, the middle line indicates the median, the edges of the box represent the first and third quartiles, and whiskers extend to span a 1.5 interquartile range from the edges. Source data are provided as a Source Data file.

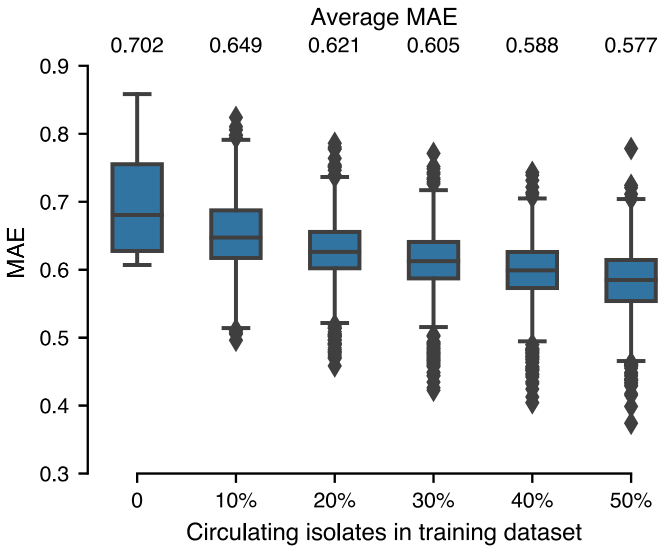

Supplementary Fig. 7 Performance of the model when partial antigenic information of circulating virus isolates is available. The model was trained on dataset consisting of genetic and antigenic information of historical isolates as well as x% of randomly selected circulating isolates, where x was varied from 10% to 50%. The simulations were repeated for 50 Monte Carlo runs. The MAE performance of the model was computed for 14 test seasons from 2014NH to 2020SH, where the average MAE over these seasons is mentioned above each boxplot. In each box plot, the middle line indicates the median, the edges of the box represent the first and third quartiles, and whiskers extend to span a 1.5 interquartile range from the edges. Source data are provided as a Source Data file.

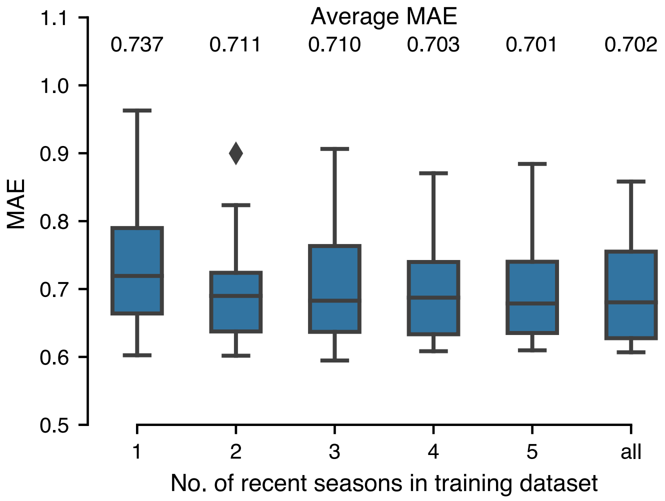

Supplementary Fig. 8 Performance of models trained over subsets of training data containing only one to five most recent seasons. For reference, 'all' denotes the case when the complete training dataset is used. Each boxplot shows the variation in the MAE performance of the models over 14 test seasons from 2014NH to 2020SH, and the average MAE over these seasons is mentioned above each boxplot. For each test season s, the x recent seasons represents the case when the model was trained over a subset of training data consisting of x seasons starting from season $s-x$ to season $s-1$. In each box plot, the middle line indicates the median, the edges of the box represent the first and third quartiles, and whiskers extend to span a 1.5 interquartile range from the edges. Source data are provided as a Source Data file.

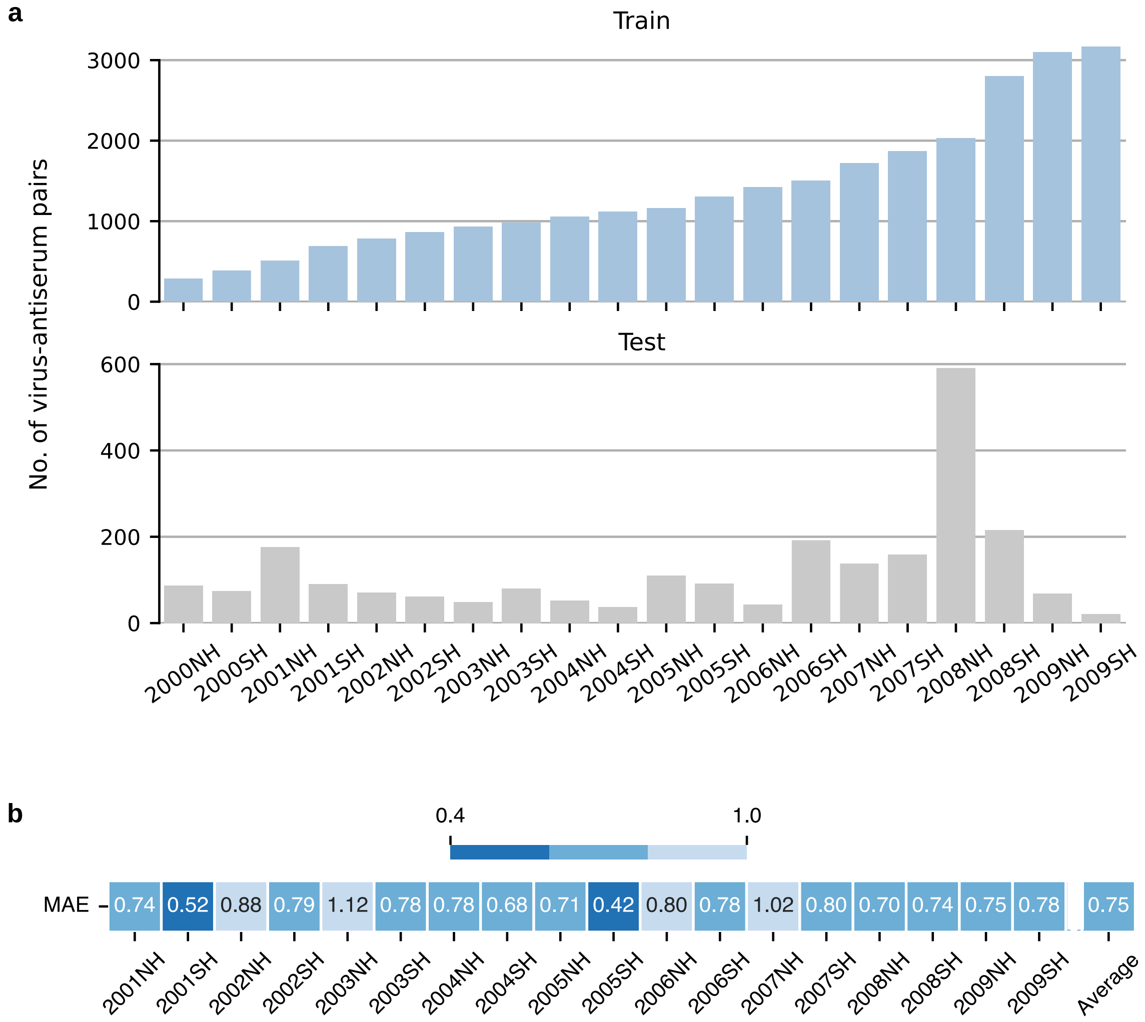

Supplementary Fig. 9 Data distribution and performance of the AdaBoost model for seasonal antigenic prediction of IAV H1N1 over multiple influenza seasons. (a) Under the seasonal framework (**Fig. 1a**), the IAV H1N1 data[35] is distributed into training and test datasets for each of the 18 seasons from 2001NH to 2009SH. The upper panel shows the number of virus-antiserum pairs in the training dataset for each season, whereas the lower panel depicts the same for the test dataset. This dataset includes a total of 3,233 NHTs computed using 18,211 HI titre values after removing threshold values from the dataset used in ref.[33]. Corresponding to this dataset, a total of 506 HA sequences were obtained from GISAID[13] using the isolate IDs provided in ref.[35] and then aligned using MAFFT[48] with reference to A/Fujian/156/2000. **(b)** The MAE performance of the AdaBoost model for seasonal antigenic prediction of IAV H1N1 over 18 seasons from 2001NH to 2009SH. The 'Average' cell indicates the score averaged over these 18 seasons. The darker coloured cells indicate better performance. The AdaBoost model (with same hyperparameters as used for IAV H3N2) was used (see *Methods*). The HA1 sequences (length 326 from amino acid position 18 to 343 of HA protein) of IAV H3N2 virus-antiserum pairs were encoded using the same mutation matrix that performed the best for IAV H3N2 (see *Methods*). As this dataset lacks the passage information, a unique virus isolate was identified by only its name, and hence the used metadata information only included virus avidity and antiserum potency estimated from corresponding names of virus isolates. Source data are provided as a Source Data file.

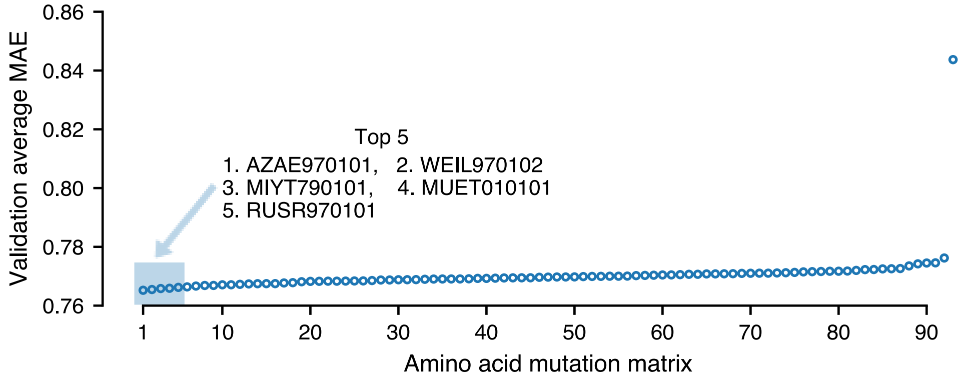

Supplementary Fig. 10 Variation in the MAE performance of the RF model for different mutation matrices. The hyperparameters were optimized to minimize the average MAE over four validation seasons (2012NH to 2013SH), independently for each RF model corresponding to the mutation matrices and binary encoding, where each model was implemented under seasonal framework (**Fig. 1**). Source data are provided as a Source Data file.

SUPPLEMENTARY TABLES

Supplementary Table 1 Hyperparameters, corresponding distribution of search space, and their optimal values for *the compared* models. (a*)* RF model (RandomForestRegressor under module Scikit-learn). **(b)** XGBoost model (XGBRegressor under module XGBoost). **(c)** MLP model (built using Keras). **(d)** ResNet model (built using Keras). The optimal values of hyperparameters for each model correspond to the top performing mutation matrix, which is ‘AZAE970101’ for the RF model, GIAG010101 for XGBoost, WEIL970102 for MLP, and MUET010101 for ResNet.

a

Hyperparameter	Search space	Optimal value
n_estimators	QUniformInt [50, 1000, 25]	125
max_features	Uniform [0.1, 0.75]	0.375553860442328
max_depth	QUniformInt [50, 200, 10]	200
min_samples_leaf	QUniformInt [1, 5, 1]	1
min_samples_split	QUniformInt [2, 30, 1]	10
bootstrap	Choice [True, False]	True

b

Hyperparameter	Search space	Optimal value
n_estimators	UniformInt [10, 500]	343
max_depth	UniformInt [2, 100]	23
subsample	Uniform [0.1, 1]	0.790391730792872
learning_rate	Uniform [0.001, 1]	0.0586498853490469
colsample_bylevel	Uniform [0.1, 1]	0.360570017142831
Colsample_bytree	Uniform [0.1, 1]	0.829414276718852

c

Hyperparameter	Search space	Optimal value
learning_rate	LogUniform [1e-5, 1e-1]	0.0000168309492546526
epochs	UniformInt [10, 200, 10]	160
# hidden layers	UniformInt [1, 5]	2
# units (hidden layer/s)	UniformInt [100, 5000, 100]	[5000, 3100]
dropout (hidden layer/s)	Uniform [0.0, 0.5, 0.1]	[0.4, 0.5]

d

Hyperparameter	Search space	Optimal value
learning_rate	LogUniform [1e-5, 1e-1]	0.003494896818018
epochs	UniformInt [10, 200, 10]	140
# units (linear layer)	UniformInt [100, 5000, 100]	3200
# ResNetBlocks	UniformInt [1, 5]	1
# units (ResNetBlock/s)	UniformInt [100, 5000, 100]	1500
dropout (ResNetBlock/s)	Uniform [0.0, 0.5, 0.1]	0.4
Residual dropout (ResNetBlock/s)	Uniform [0.0, 0.5, 0.1]	0
