# Supplementary material for: Seasonal antigenic prediction of influenza A H3N2 using machine learning
Source: Nat Commun. 2024 May 7;15:3833. doi: 10.1038/s41467-024-47862-9 (PMC11076571; doi:10.1038/s41467-024-47862-9)
Supplement: Supplementary file 3 — Description of Additional Supplementary Files [file 41467_2024_47862_MOESM3_ESM.docx]

Description of additional supplementary files

**Title:** Supplementary Data 1.

**Description:** Antigenic and genetic information of IAV H3N2 from 2003 – 2021. This supplementary data file includes the influenza A virus H3N2 antigenic data of influenza seasons from 2003 – 2021 published in 35 biannual reports by the Worldwide Influenza Centre at the Francis Crick Institute. It also includes the matched HA protein sequence information virus and antiserum from genetic databases, GISAID and IVR. This dataset comprises 53,176 data samples corresponding to valid haemagglutination inhibition (HI) titre values and egg or cell passage category information against virus and antiserum in the Crick reports (see *Methods* for further details). It contains the following 18 column headers:

| **Column header** | **Description** |
| --- | --- |
| virusName | name of virus |
| virusPassage | passage of virus |
| virusPassCat | passage category of virus |
| virusDate | collection date of virus |
| virusGroup | genetic group/clade of virus |
| serumName | name of antiserum |
| serumPassage | passage of antiserum |
| serumPassCat | passage category of antiserum |
| serumDate | collection date of virus against which antiserum is raised |
| serumGroup | genetic group/clade of antiserum |
| ferret | ferret identifier against which antiserum is collected |
| source | source of the corresponding data sample |
| virusSeqDB | name of genetic database for virus sequence |
| virusSeqID | accession number of virus sequence |
| virusMatchedPass | virus passage from genetic database |
| serumSeqDB | name of genetic database for antiserum sequence |
| serumSeqID | accession number of antiserum sequence |
| serumMatchedPass | antiserum passage from genetic database |
