# Supplementary material for: Seasonal antigenic prediction of influenza A H3N2 using machine learning
Source: Nat Commun. 2024 May 7;15:3833. doi: 10.1038/s41467-024-47862-9 (PMC11076571; doi:10.1038/s41467-024-47862-9)
Supplement: Supplementary file 5 — Reporting Summary [file 41467_2024_47862_MOESM5_ESM.pdf]

nature portfolio

Corresponding author(s): Matthew R. McKay and Ahmed A. Quadeer

Last updated by author(s): Mar 19, 2024

Reporting Summary

Nature Portfolio wishes to improve the reproducibility of the work that we publish. This form provides structure for consistency and transparency in reporting. For further information on Nature Portfolio policies, see our Editorial Policies and the Editorial Policy Checklist.

Statistics

For all statistical analyses, confirm that the following items are present in the figure legend, table legend, main text, or Methods section.

n/a	Confirmed	
☐	☒	The exact sample size (*n*) for each experimental group/condition, given as a discrete number and unit of measurement
☐	☒	A statement on whether measurements were taken from distinct samples or whether the same sample was measured repeatedly
☐	☒	The statistical test(s) used AND whether they are one- or two-sided<br>*Only common tests should be described solely by name; describe more complex techniques in the Methods section.*
☒	☐	A description of all covariates tested
☐	☒	A description of any assumptions or corrections, such as tests of normality and adjustment for multiple comparisons
☐	☒	A full description of the statistical parameters including central tendency (e.g. means) or other basic estimates (e.g. regression coefficient) AND variation (e.g. standard deviation) or associated estimates of uncertainty (e.g. confidence intervals)
☐	☒	For null hypothesis testing, the test statistic (e.g. *F*, *t*, *r*) with confidence intervals, effect sizes, degrees of freedom and *P* value noted<br>*Give P values as exact values whenever suitable.*
☒	☐	For Bayesian analysis, information on the choice of priors and Markov chain Monte Carlo settings
☐	☒	For hierarchical and complex designs, identification of the appropriate level for tests and full reporting of outcomes
☒	☐	Estimates of effect sizes (e.g. Cohen's *d*, Pearson's *r*), indicating how they were calculated

Our web collection on statistics for biologists contains articles on many of the points above.

Software and code

Policy information about availability of computer code

Data collection	Tabula 1.2.1 was used to extract HI titre tables for influenza A virus (IAV) H3N2 from Crick Worldwide Influenza Centre (WIC) reports.
Data analysis	All statistical analyses in this work were performed using Python 3.8.12.<br>Scripts for reproducing the results are available at https://github.com/saws-lab/SAP_H3N2_ML.<br>Streamlit (https://streamlit.io) was used to develop the web application for seasonal antigenic prediction of IAV H3N2 using our proposed AdaBoost model, which can be accessed from Hugging Face Spaces at https://huggingface.co/spaces/sawshah/SAP_H3N2.<br>To observe antigenic drift of IAV H3N2 isolates across seasons, we performed antigenic cartography of these isolates using R's (version 4.2.0) Racmacs package (version 1.1.35).<br>For computing the distance between atoms in the HA protein structure and for drawing the structural figures, the PyMOL software (https://www.pymol.org) was used.

For manuscripts utilizing custom algorithms or software that are central to the research but not yet described in published literature, software must be made available to editors and reviewers. We strongly encourage code deposition in a community repository (e.g. GitHub). See the Nature Portfolio guidelines for submitting code & software for further information.

Data

Policy information about availability of data

All manuscripts must include a data availability statement. This statement should provide the following information, where applicable:

- Accession codes, unique identifiers, or web links for publicly available datasets
- A description of any restrictions on data availability
- For clinical datasets or third party data, please ensure that the statement adheres to our policy

The antigenic HI titre data for IAV H3N2 were obtained from biannual reports published by Worldwide Influenza Centre at the Francis Crick Institute, London at https://www.crick.ac.uk/research/platforms-and-facilities/worldwide-influenza-centre/annual-and-interim-reports. The antigenic HI titre data for IAV H1N1 were obtained from the published dataset at https://researchdata.gla.ac.uk/289. The corresponding HA protein sequences for IAV H3N2 and H1N1 were downloaded from the GISAID (https://gisaid.org) and the IVR (https://www.ncbi.nlm.nih.gov/genomes/FLU/Database/nph-select.cgi?go=database) databases. Supplementary Data 1 provides the information of the virus-antiserum pairs of IAV H3N2 used in this analysis. It identifies the specific HI data from the Crick WIC reports and the HA protein sequence data from the GISAID and the IVR databases. The three-dimensional HA structure of IAV H3N2 A/Brisbane/10/2007 (PDB ID: [6AOU] https://doi.org/10.2210/pdb6AOU/pdb) used in this analysis was obtained from the Protein Data Bank (https://www.rcsb.org). All data used in this work is publicly available as of the date of publication. Source data for all figures are provided with this paper. Any additional information related to the data reported in this paper is available from the lead contact upon request.

Research involving human participants, their data, or biological material

Policy information about studies with human participants or human data. See also policy information about sex, gender (identity/presentation), and sexual orientation and race, ethnicity and racism.

Reporting on sex and gender	This study does not involved any sex or gender information as this information does not influence the conclusions drawn by the analysis conducted in the study.
Reporting on race, ethnicity, or other socially relevant groupings	N/A
Population characteristics	All IAV H3N2 and H1N1 sequences anlalyzed in this study were collected from patients infected with the respective IAV H3N2 and H1N1 viruses.
Recruitment	N/A
Ethics oversight	N/A

Note that full information on the approval of the study protocol must also be provided in the manuscript.

Field-specific reporting

Please select the one below that is the best fit for your research. If you are not sure, read the appropriate sections before making your selection.

☐ Life sciences ☐ Behavioural & social sciences ☒ Ecological, evolutionary & environmental sciences

For a reference copy of the document with all sections, see nature.com/documents/nr-reporting-summary-flat.pdf

Ecological, evolutionary & environmental sciences study design

All studies must disclose on these points even when the disclosure is negative.

Study description	We have presented a machine learning model that can accurately predict antigenic properties (in terms of NHTs) of IAV H3N2 isolates circulating in an influenza season using only their genetic sequence data and associated metadata.
Research sample	The antigenic HI titre data for IAV H3N2 were obtained from biannual reports published 2003 – 2021 by Worldwide Influenza Centre at the Francis Crick Institute, London. A total of 82,776 HI titre values against virus-antiserum pairs were extracted from these reports. The antigenic HI titre data for IAV H1N1 were obtained from the published dataset at https://researchdata.gla.ac.uk/289. In total, 48,707 HI titers measured between 29,600 unique combinations of virus and antiserum are present in the H1N1 dataset. The corresponding HA protein sequences for 3746 IAV H3N2 viruses and 506 IAV H1N1 viruses were downloaded from the GISAID and the IVR databases.
Sampling strategy	For IAV H3N2, we preferred the sequences with complete HA1 subunit and correctly matched metadata. For IAV H1N1, we used the sequences already provided with the dataset.
Data collection	The antigenic HI titre data for IAV H3N2 were obtained from biannual reports published by Worldwide Influenza Centre at the Francis Crick Institute, London at https://www.crick.ac.uk/research/platforms-and-facilities/worldwide-influenza-centre/annual-and-interim-reports. The antigenic HI titre data for IAV H1N1 were obtained from the published dataset at https://researchdata.gla.ac.uk/289. The corresponding HA protein sequences for IAV H3N2 and H1N1 were downloaded from the GISAID (https://gisaid.org) and the IVR (https://www.ncbi.nlm.nih.gov/genomes/FLU/Database/nph-select.cgi?go=database) databases.

Timing and spatial scale	The antigenic HI titre data for IAV H3N2 from the Crick WIC biannual reports were periodically downloaded between 2020-08-08 to 2023-03-10. The antigenic HI titre data for IAV H1N1 was downloaded once on 2023-10-27. The HA protein sequences for IAV H3N2 were periodically downloaded from GISAID and IVR between 2020-12-23 to 2023-04-04. The HA protein sequences for IAV H1N1 were downloaded from GISAID on 2023-10-29. The collection dates of the HA protein sequences were obtained from GISAID and IVR and provided in Supplementary Data 1.
Data exclusions	We removed the virus-antiserum pairs from antigenic HI data against which sequences were not found in the influenza genetic databases and used the remaining antigenic data for seasonal antigenic characterization. We also removed virus-antiserum pairs with passage category other than cell or egg. For IAV H1N1, we removed threshold HI titre values and only considered HI titre values used in reference https://doi.org/10.1371/journal.ppat.1005526.
Reproducibility	Scripts for reproducing the results are available at https://github.com/saws-lab/SAP_H3N2_ML.
Randomization	This is not relevant to this study, as all data has been used to infer the model and conduct the analysis.
Blinding	This is not relevant to this study, as all data has been used to infer the model and conduct the analysis.

Did the study involve field work? ☐ Yes ☒ No

Reporting for specific materials, systems and methods

We require information from authors about some types of materials, experimental systems and methods used in many studies. Here, indicate whether each material, system or method listed is relevant to your study. If you are not sure if a list item applies to your research, read the appropriate section before selecting a response.

Materials & experimental systems

n/a	Involved in the study
☒	☐ Antibodies
☒	☐ Eukaryotic cell lines
☒	☐ Palaeontology and archaeology
☒	☐ Animals and other organisms
☒	☐ Clinical data
☒	☐ Dual use research of concern
☒	☐ Plants

Methods

n/a	Involved in the study
☒	☐ ChIP-seq
☒	☐ Flow cytometry
☒	☐ MRI-based neuroimaging

Plants

Seed stocks	*Report on the source of all seed stocks or other plant material used. If applicable, state the seed stock centre and catalogue number. If plant specimens were collected from the field, describe the collection location, date and sampling procedures.*
Novel plant genotypes	*Describe the methods by which all novel plant genotypes were produced. This includes those generated by transgenic approaches, gene editing, chemical/radiation-based mutagenesis and hybridization. For transgenic lines, describe the transformation method, the number of independent lines analyzed and the generation upon which experiments were performed. For gene-edited lines, describe the editor used, the endogenous sequence targeted for editing, the targeting guide RNA sequence (if applicable) and how the editor was applied.*
Authentication	*Describe any authentication procedures for each seed stock used or novel genotype generated. Describe any experiments used to assess the effect of a mutation and, where applicable, how potential secondary effects (e.g. second site T-DNA insertions, mosiacism, off-target gene editing) were examined.*
